# Cinnamon supplementation in broiler and laying hen diets: benefits, mechanisms, supplementation doses, and limitations - a review

**DOI:** 10.1007/s11250-026-05302-4

**Published:** 2026-11-02

**Authors:** I. P. Ogbuewu, C. A. Mbajiorgu

**Affiliations:** 1 Department of Animal Science and Technology, Federal University of Technology, P.M.B. 1526, Owerri, Imo State Nigeria; 2 Department of Agriculture and Animal Health, University of South Africa, Florida Science Campus, Private Bag X6, Florida, 1710 South Africa

**Keywords:** Cinnamon, Broilers, Layers, Growth performance, Egg production, Egg quality, Meat quality, Supplementation dosage

## Abstract

Cinnamon and its derivatives (bark powder, essential oil, extract, and nanoparticles) were used as phytogenic feed additives (PFAs) in the modern poultry industry possibly due to their antioxidant, antimicrobial, anti-inflammatory, and gut-promoting properties. The present review pools recent studies on the effects of cinnamon supplementation on broiler and laying hen performance, focusing on growth performance, carcass and meat quality, egg production, egg quality, and hatchability. Cinnamon bioactive compounds, particularly cinnamaldehyde, has been show to improve nutrient utilization through multiple mechanisms of action: direct stimulation of digestive enzymes, enhancement of intestinal morphology, and selective suppression of pathogenic bacteria and proliferation of non-pathogenic bacteria. In broilers, cinnamon supplementation has been indicated to enhance body weight gain (BWG), feed conversion efficiency (FCE), carcass yield, and meat quality traits, including water holding capacity (WHC), meat tenderness, oxidative capacity, and sensory attributes. In laying hens, studies suggest that cinnamon improve egg production, egg mass, internal egg quality, shelf life, and reduce cholesterol content in egg yolk. Supplementation doses are highly form-and context-dependent: 0.2–0.5% cinnamon bark powder (CBP) and 0.2–0.4% extract for broilers under thermoneutral conditions. For laying hens, evidence suggests an effective range of 0.75% to 1.0% CBP, while cinnamon essential oil (CEO) level remain less defined. Under heat stress conditions, 0.03–0.06% CEO or 0.06% cinnamon nanoparticles indicate superior performance. However, results of the effects of cinnamon supplementation on broiler and laying hen performance were inconsistent due to variation in dosage, cinnamon form, bird strain, age, and environmental conditions. High doses of (exceeding 2–3% CBP or 0.01% cinnamaldehyde) can reduce feed intake, elevate blood aspartate aminotransferase (AST) levels, and disrupt cecal microbiota balance. While cinnamon shows promise as a PFA in poultry nutrition, several challenges, including dose optimization, thermal stability in tropical climates, palatability, and long-term residue safety, require further research. Standardized, region-specific studies are also required to facilitate evidence-based commercial adoption of cinnamon in sustainable poultry production.

## Introduction

The poultry industry is one of the fastest-growing agricultural sectors globally, driven by rising demand for chicken meat and eggs (Anghelescu 2025; Nassar 2026). Global poultry meat consumption has grown steadily over the past decade and is projected to increase substantially by 2050 (OECD/FAO 2021). According to Wood et al. (2025) and OECD/FAO (2023), global egg production is projected to rise by 12% between 2022 and 2032, with 76% of production occurring in low- and middle-income countries (LMICs), where both populations and income levels are rising rapidly. In this context, promoting sustainable intensive poultry production, improving feed conversion efficiency (FCE), and consistently delivering safe, high-quality products to consumers will be essential to meeting future demand. Phytogenic feed additives (PFAs), which are additives derived from herbs, spices, and essential oils, have gained considerable research attention due to their likely ability to improve feed intake, FCE, nutrient utilization, immunity, and overall health in chickens (Abdelli et al. 2021; Chen et al. 2022; Ogbuewu and Mbajiorgu 2023; Liu et al. 2023; Rasheed et al. 2024; Al-Garadi et al. 2025).

Among these PFAs, cinnamon and its derivatives, including bark, fruit, essential oil, and leaves have shown promise as a feed additive in poultry production owing to their growth - and health-promoting effects of their bioactive compounds (Qaid et al. 2022; Fathi et al. 2025a; b; Fathi and Zarrinkavyani 2026). Cinnamon is an aromatic herb native to Sri Lanka and southern India that belongs to the Lauraceae family. Although over 300 species of cinnamon have been identified globally, *Cinnamomum verum* and *C. zeylanicum* are the most promising for animal feeding due to their rich bioactive compound profiles. Cinnamon has a long history of use in traditional medicine for treating various ailments (Bansal et al. 2025). The bark is particularly rich in bioactive compounds, including eugenol, cinnamate, cinnamic acids, linalool, camphor, cinnamaldehyde, cinnamyl acetate, and cinnamic acid. These compounds contribute to improved poultry health and performance (Hamidpour et al. 2015; Ali et al. 2021) by exerting antioxidant, anti-inflammatory, antimicrobial, and gut-health-promoting effects in animals (Wang et al. 2020a; Pathak et al. 2021).

Numerous feeding studies have reported the beneficial effects of cinnamon supplementation on growth performance, blood parameters, intestinal health, egg production, and egg quality in chickens (Ali et al. 2021; Ghanem et al. 2021; Kang et al. 2024; Fathi et al. 2025a; c). However, the results remain inconsistent. While some studies demonstrate significant improvements (Qaid et al. 2022; Jimoh et al. 2024a; Sompie et al. 2024), others report no significant effect (Cifti et al. 2009; Sompie et al. 2024; Elal Mus et al. 2026) or even negative outcomes at certain doses and forms (Abo Ghanima et al. 2020; Qaid et al. 2022). These discrepancies are largely attributed to variations in supplementation dosages, cinnamon form (powder, essential oil, extracts, or nanoparticles), bird strain, age, housing conditions, and basal diet composition.

This review, therefore, synthesizes recent studies on the mechanisms, benefits, limitations, and safety concerns associated with cinnamon supplementation in broiler and laying hen production. It also identifies cinnamon dosage ranges based on consistency of outcome and highlights existing knowledge gaps to facilitate the effective and safe adoption of cinnamon in the poultry industry.

## Methodology

**Fig. 1:**
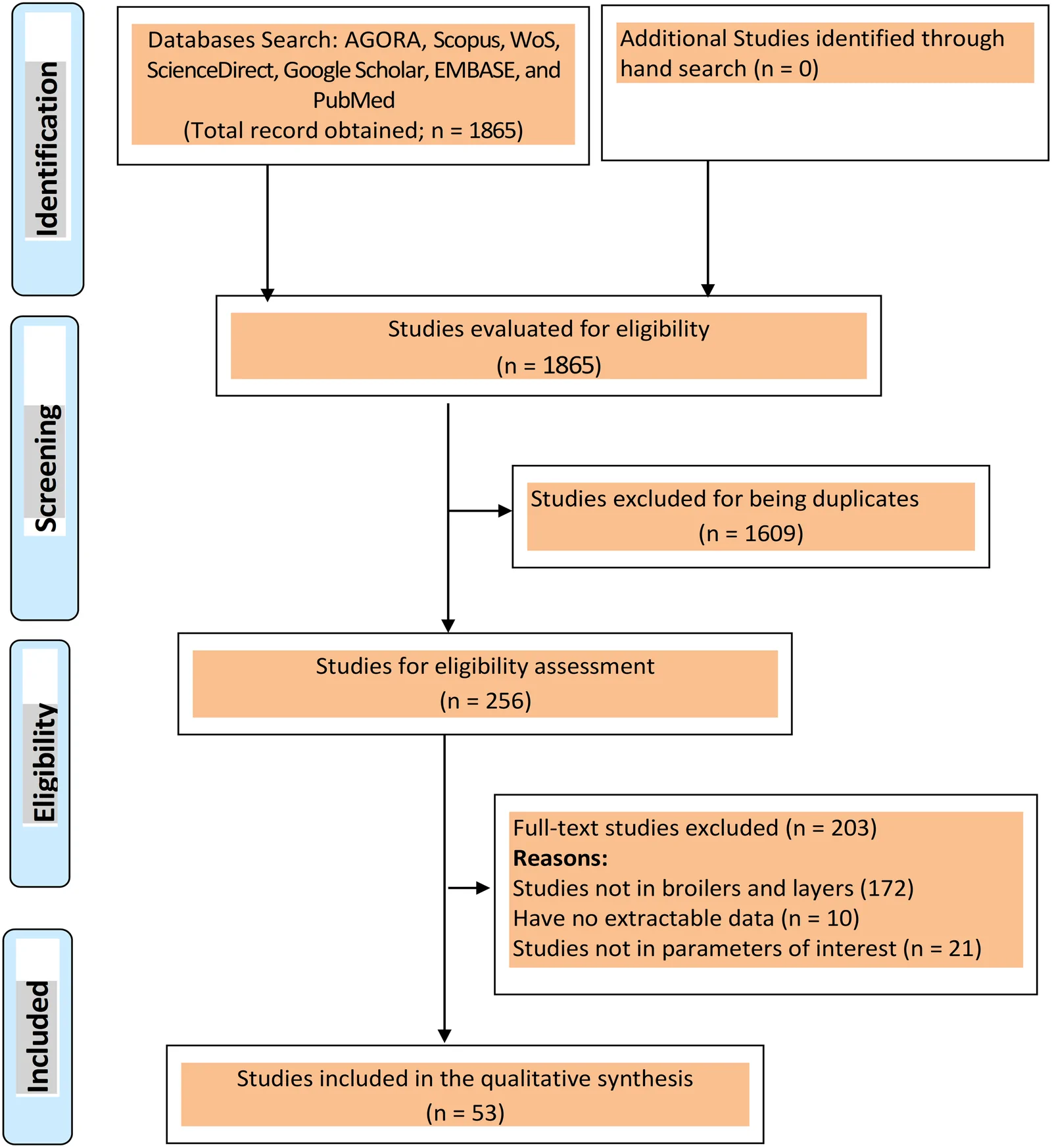
Flowchart of selection process for the review

This review was performed using studies retrieved from seven electronic databases: Google Scholar, Scopus, PubMed, AGORA, Web of Science, EMBASE, and ScienceDirect. These databases were searched between 4th and 10th April 2026 with no restriction placed on publication language and date. The keywords used included: cinnamon, CEO, CBP, cinnamon powder, cinnamon extract, cinnamaldehyde, chemical composition, antioxidant, mechanisms of action, poultry, broiler chickens, laying hens, and layers. Other keywords included were growth performance, laying rate, egg production, egg quality, hatchability, palatability, carcass, meat quality, blood parameters, microbiota, gut health, economics of production, and safety concerns. These keywords were combined using Boolean operators (AND, OR) and wildcards (*). The inclusion criteria were as follows: studies that evaluated the chemical composition of cinnamon, its mechanisms of action, or the effects of cinnamon supplementation on the performance of broilers and laying hen. Exclusion criteria included: (i) studies not conducted in broilers or laying hens; (ii) studies with no extractable data; and (iii) studies that measured parameters not relevant to the review topic. The search identified 1865 articles, which were initially imported into Zotero management reference software (Version 8.0.5) for duplicate identification and removal before being for screening. After removing 1609 duplicates by merging them, 256 articles remained. Titles and abstracts were independently screened by two independent reviewers based on the predefined eligibility criteria, followed by full-text assessment of potentially relevant articles. Of the 256 articles left, 203 were excluded. A total of 53 studies were included in the review as shown in Fig. 1.

### Quality assessment

All the included studies were subjected to risk-of-bias assessment across domains using SYRCLE’s Risk of Bias tool as presented in Table 1. All assessments were performed independently by two reviewers, with disagreements resolved through consensus to ensure consistency and reliability. The methodological quality of the evidence base is therefore moderate to low, primarily due to poor reporting and inadequate experimental design in key. The included studies showed unclear or high risk of bias across most domains. Incomplete outcome data showed excellent performance (94% low risk). Attrition bias is minimal, and indicating that most studies handled missing data appropriately. Other bias revealed strong performance (85% low risk), indicating few additional sources of bias beyond the assessed domains. The fact that researchers did not usually report in detail how they allot bird to pens resulted in an unclear risk of bias for sequence generation. Consequently, it was impossible to confirm that all outcomes had been reported. In addition, the randomization methods were rarely described in sufficient detail. Some studies show acceptable methodological quality in some key domains. However, future studies in this field should prioritize transparent reporting, proper randomization procedures, allocation concealment, and blinding.

**Table 1:** Summary of risk-of-bias assessment

Domain	Low risk	Unclear risk	High risk	% low risk
1. Sequence generation	0	53	0	0%
2. Baseline characteristics	0	53	0	0%
3. Allocation concealment	2	51	0	4%
4. Random housing	3	50	0	6%
5. Blinding (attendants)	0	13	40	0%
6. Random outcome assessment	0	53	0	0%
7. Blinding (assessors)	5	48	0	9%
8. Incomplete outcome data	50	3	0	94%
9. Selective reporting	0	53	0	0%
10. Other bias	45	8	0	85%

### Chemical constituents of cinnamon

Cinnamon is commercially available in various forms for use in poultry production, including bark, essential oil, extract, and nanoparticles (Jimoh et al. 2024a; Ibigbami et al. 2025; Fathi et al. 2025a). Each form has distinct chemical compositions and bioavailability characteristics. Cinnamon bark is the most traditional form and contains 3.3–4.43% crude protein (CP), 24.4–31.0% crude fiber (CF), 54.4–64.0% carbohydrates, 2.2–3.18% ash, and 3.8–4.03% ether extract (EE) on a dry matter (DM) basis (Farrukh 2019; Qaid et al. 2025). Fiber analysis by Qaid et al. (2025) found that CBP contains 45.8% acid detergent fiber (ADF) and 65.3% neutral detergent fiber (NDF), indicating that CBP that it is rich in cell wall biomass. Farrukh (2019) also reported high mineral content in cinnamon bark, including 134.7 mg/g potassium, 83.8 mg/g calcium, 20.1 mg/g manganese, 42.4 mg/g phosphorus, 7.2 mg/g iron, and 2.6 mg/g zinc. These findings align with Goel and Mishra (2020), who highlighted cinnamon as a valuable source of both micro and macronutrients. Cinnamon essential oil (CEO) is rich in bioactive compounds and may require lower supplementation levels than cinnamon bark to achieve comparable effects. These bioactive compounds, including cinnamaldehyde, β-caryophyllene, cinnamic acid, cinnamate, eugenol, carvacrol, linalool, camphor, and cinnamyl acetate vary depending on the plant part used (bark, leaf, and powder). (Farrukh 2019; Ali et al. 2021; Zhao et al. 2022; Hongsheng et al. 2023). As shown in Table 2, cinnamon leaf is high in eugenol and phenylpropanoids, while roots and fruits are predominantly rich in monoterpenes and sesquiterpenes, respectively. In contrast, the bark contains cinnamaldehyde and phenylpropanoids, which are primarily responsible for its potent antimicrobial, antioxidant, and anti-inflammatory properties (Pagliari et al. 2023; Gębarowska et al. 2026). Furthermore, studies by Saeed et al. (2018) and Zhao et al. (2022) indicate that cinnamon products contain polyphenols, flavonoids, tannins, phenolic acids, saponins, coumarin, choline, carbohydrates, various vitamins (retinol, phylloquinone, ascorbic acid, and niacin), and minerals such as potassium, calcium, iron, and magnesium.

**Table 2:** Chemical composition of different parts of *C. verum*

Compounds	Percentage (%)			
	Leaf	Fruit	Root	Bark
Eugenol	76.74	4.15	0.21	4.15
Pinene	0.73	2.19	5.70	3.34
Phenylpropanoids	85.40	0.90	2.20	64.8
Cymene	0.92	0.01	1.38	1.91
Linalool	2.77	0.08	0.13	3.70
Monoterpenes	6.70	6.70	95.2	25.3
Limonene	0.30	1.00	6.20	1.20
Sesquiterpenes	3.47	83.6	0.70	8.70
Terpinolene	0.61	0.30	0.47	0.21
Cinnamaldehyde	2.70	0.30	0.10	50.5
Cryophyllene	3.47	5.63	0.62	8.00
Cinnamy-acetate	1.00	0.10	0.12	8.78

***Adapted from*** Farrukh (2019)

### Mechanism of action of cinnamon

Research indicates that cinnamon may enhance poultry performance through complex, multifactorial biological mechanisms that improve small intestine absorptive capacity, antimicrobial defense, antioxidant status, and immune function (Saeed et al. 2018). These beneficial effects may be driven by its key bioactive components, including cinnamaldehyde, eugenol, phenylpropanoids, sesquiterpenes, and monoterpenes (Farrukh 2019). Cinnamaldehyde, the main bioactive compound in cinnamon bark, inhibits the proliferation of pathogenic microbes in the chicken intestine (Yang et al. 2021a; Jimenez et al. 2024). Its possible antibacterial mechanisms include: (1) binding to the bacterial cell surface and disrupting the integrity of the cell membrane phospholipid bilayer, (2) inhibition of ATP production, (3) inhibition of peptidoglycan synthesis or protein denaturation, and (4) disturbance of cell membrane structural integrity. These actions may impair cellular metabolism, resulting in cell death (Zheng et al. 2020; Sobhy et al. 2023).

Another likely mechanism is cinnamon’s potent antioxidant benefits (Elashry et al. 2026; Fathi and Zarrinkavyani 2026). Studies indicate that cinnamon supplementation enhance antioxidant defense in poultry by increasing the activity of endogenous enzymes such as superoxide dismutase (SOD), catalase (CAT), and glutathione peroxidase (GPx), while lowering malondialdehyde (MDA) levels, a key biomarker of lipid peroxidation (Elashry et al. 2026; Fathi and Zarrinkavyani 2026). Additionally, dietary cinnamon has been shown to upregulate the expression of mucin 2 (MUC2), heat shock protein 70 (HSP70), and the tight junction protein zona occludens-1 (ZO-1) in broilers, which improve intestinal barrier integrity, mucus protection, and cellular stress resilience (Shehata et al. 2020; Sampath et al. 2025). Cinnamon bioactive compounds have also been demonstrated to enhance systemic immunity while lowering MDA content (Jimoh et al. 2024a).

Long-term inflammation redirects energy and nutrients away from growth and production toward immune defense (Lacourt et al. 2018). Cinnamon may help to counteract this effect by suppressing inflammation through the reduction of pro-inflammatory cytokines such as interleukin-1β (IL-1β) and interferon-γ (IFN-γ), while increasing the anti-inflammatory interleukin-10 (IL-10). This modulation conserves energy and nutrients for productive functions in chicken (Jimoh et al. 2024a; Elashry et al. 2026). In addition, cinnamon enhance nutrient utilization by stimulating the digestive enzyme secretion, including protease, amylase, and lipase, thereby improving digestion and absorption (Fathi et al. 2025b). It also increases feed intake and FCE by enhancing villus structure and upregulating genes involved in nutrient transport (Kang et al. 2024; Elashry et al. 2026). Furthermore, its bioactive compounds exert lipid-and cholesterol-lowering effects by regulating lipid metabolism (Abo Ghanima et al. 2020).

### Effect of cinnamon on gut health

The ban on the use of antibiotics in animal feed as a growth promoters has been driven by increasing antimicrobial resistance and intense consumer demand for antibiotic-free products. This has led to the search for safe and effective alternatives that can support chicken health, particularly gastrointestinal (gut) health, which is central to nutrient absorption and immune function (Ali et al. 2021). Dietary cinnamon supplementation has been reported to enhance intestinal morphology [long villi height (VH) and high VH – to crypt depth (CD) ratio], which is vital for improving nutrient absorption. A study by Qaid et al. (2022) revealed that broilers fed diets having 0.2% CBP had higher duodenal VH, VH/CD ratio, and goblet density than those offered diets without CBP supplementation. Similarly, broilers on 0.3% and 0.6% dietary CBP intervention showed increased intestinal VH, CD, and muscle thickness, suggesting improved absorptive capacity (Ibigbami et al. 2025; Elashry et al. 2026). Supplementation at 0.4% CEO in broiler diets also improves VH of the duodenum and jejunum (Doneria et al. 2022). These structural improvements can l; ead to higher nutrient absorption and enhanced growth performance.

Cinnamon has been shown to reduce populations of *Campylobacter jejuni* and pathogenic *Escherichia coli* while increasing the population of beneficial *Lactobacillus* species in disease-challenged broilers (Ali et al. 2021). This demonstrates its potential to mitigate enteric infections and promote a balanced, healthy gut microbes. Broiler infected with *Salmonella pullorum* show distinct signs of disease, including activation of intestinal epithelial cells and recruitment of macrophages, neutrophils, and eosinophils into the intestinal lumen (Wang et al. 2020b). The ability of cinnamon-supplemented diets to suppress the proliferation of pathobionts, while promoting the growth of beneficial bacteria can improve intestinal health. This action, can in turn, strengthens resistance to pathogen colonization, reduces inflammatory burden, and help maintain a stable intestinal ecosystem. Cinnamon, particularly its main bioactive cinnamaldehyde, strengthens intestinal barrier integrity by upregulating the mRNA expression of key tight-junction proteins, including claudin-1, occludin-1, and ZO-1. This reduces intestinal permeability and prevents translocation of luminal pathogens, toxins, and antigens (Sampath et al. 2025; Yin et al. 2025).

Doneria et al. (2022) reported that dietary CEO supplementation improves immune response in broilers, as shown by higher antibody titers against viral pathogens. Additionally, the anti-inflammatory effects of cinnamon can be a key contributor to its gut health benefits. Studies have shown that dietary supplementation with cinnamaldehyde, the major bioactive compound in cinnamon, reduces the levels of pro-inflammatory cytokines, such as interleukin-1 beta (IL-1β) and tumor necrosis factor alpha (TNF-α), while increasing the level of the anti-inflammatory cytokine IL-10 (Yin et al. 2025; Elashry et al. 2026). These changes help alleviate the chronic inflammatory state that can impair intestinal integrity and nutrient utilization in broilers.

### Effects of cinnamon supplementation on growth performance parameters

Cinnamon is increasingly recognized as a novel PFA in broiler production. Some studies have demonstrated its ability to enhance growth performance (Table 3) through several possible mechanisms of action (Shehata et al. 2020; Yang et al. 2021a; Sampath et al. 2025). Qaid et al. (2025) reported that broilers fed a basal diet supplemented with 0.2% CBP had no beneficial effects on feed intake, body weight gain (BWG), and FCE compared to those fed basal diets supplemented with 0.4 and 0.6% CBP. The lack of significant improvements on feed intake, FCE, and BWG in broilers that received 0.4% and 0.6% CBP contrasts with the findings of Khan et al. (2025). In that study, supplementation with 0.5 and 1.0% CBP increased BWG by 10% and 14%, feed intake by 5% and 7%, and FCE by 4 and 8%, respectively. This discrepancy may be attributed to differences in broiler strain, basal diet composition, cinnamon source or environmental conditions. The observed increases in FCE and BWG could be attributed to enhanced digestive enzyme activity, better intestinal morphology, improved nutrient utilization, and antimicrobial effects of cinnamon that can reduce pathogen competition (Fathi et al. 2025b). Similarly, Tahami et al. (2023) found that supplementation with 0.015% CBP resulted in higher BWG in broilers compared to the control group. An earlier study by Park (2008) revealed that broilers fed 3% CBP achieved higher FBW than those fed 4 and 5% CBP, suggesting that 3% may represent the optimal threshold for supporting FBW in broilers. This suggests that at 3% supplementation level, the bioactive compounds in CBP exert antimicrobial effects that reduce competition between broilers and intestinal pathogens for nutrients, consequently improving FCE and BWG (Saeed et al. 2018; Yang et al. 2021a; Kang et al. 2024).

Dietary supplementation with 1.5% CEO increased feed intake, FBW, FCE, and BWG, while reducing mortality rates in Cobb 500 male broilers exposed to cyclic heat stress from days 29 to 35 (Wang et al. 2025). These improvements in the growth performance of broilers fed cinnamon products corroborate the reports of Sampath et al. (2025), who reported that cinnamon supplementation improves the absorptive capacity of the small intestine in broilers. Under heat stress conditions (34 ± 2 °C for 8 h from days 25 to 42), supplementation with cinnamon nanoparticle (CNP) at 0.015, 0.03, 0.045, and 0.06% produced dose-dependent benefits in growth performance (Fathi and Zarrinkavyani 2026). The authors reported that FBW and BWG increased linearly with increasing CNP levels, with the highest value found in birds supplemented with 0.06% CNP compared to the heat-exposed control group. Feed conversion efficiency was also increased while mortality rate decreased (Fathi and Zarrinkavyani 2026). These findings reveal that CNP can alleviates heat stress and improves growth performance in broilers, likely through increased intestinal surface area and enhanced nutrient bioavailability. Similarly, Bosetti et al. (2020) noticed that dietary supplementation with 0.01%, 0.02%, and 0.04% of a micro-encapsulated essential oil (EO) blend containing 60% cinnamaldehyde, 30% carvacrol, and 10% carrier resulted in improved feed intake, FCE, BWG, and productive efficiency index (IPE) in broilers. This suggests that cinnamon’s bioactive compounds primarily cinnamaldehyde and eugenol, can act as natural digestive stimulants and antimicrobial agent, resulting in better growth performance in healthy broilers (Qaid et al. 2021). Also, dietary administration of cinnamon bark extract at 0.015% in broiler improved growth performance and nutrient digestibility (Khanghahi et al. 2025). In a comparative study, both cinnamon extract (CNE) and cinnamon nanoparticles at 0.02% and 0.04% increased FCE and BWG compared to the control, with the highest BWG obtained in the nanoparticle group. This indicates that while the extract form is effective as a feed additive in broiler production, the nanoparticle formulation is more potent (Fathi et al. 2025a). In contrast, Qaid et al. (2022) reported that CEO supplementation at 0.2-06% reduced BWG in broilers. Similarly, Sulaiman et al. (2024) observed reduced BWG in broilers fed diets supplemented with CBP at 0.05, 0.15, 0.20, and 0.25% from days 1–49. These discrepancies may be attributed to variations in chemical composition of cinnamon used, basal diet composition, duration of supplementation, and broiler genetics.

The superior FCE and BWG observed, especially in broilers fed a diet supplemented with CNP at 0.04% can be attributed to enhanced nutrient utilization and improved gut health. This observation agrees with previous reports by Asadi et al. (2023) showing that nanoparticle formulations can enhance the stability and bioavailability of phytochemicals, thus, leading to improved digestion and nutrient absorption. The enhanced growth performance in broilers on cinnamon nano formulation is likely attributable to increased intestinal surface area and improved nutrient solubility, which promotes efficient cellular uptake and controlled release of bioactive compounds (Anand et al. 2010). Similar benefits, including improved FCE and gut functionality have been observed in other studies on plant-based nanoparticles in broilers (Ebrahimzadeh et al. 2020; Hussein et al. 2021). Overall, results indicate that cinnamon improves growth performance possibly via one or a blend of the following mechanisms of action: direct antioxidant activity (Wang et al. 2020a), enhancement of endogenous antioxidant systems (Fathi and Zarrinkavyani 2026), gut health modulation (Yang et al. 2021a), and regulation of gene expression (Kang et al. 2024; Elashry et al. 2026). Nevertheless, nano formulation has shown potential as effective growth promoter, offering enhanced benefits under both normal and heat-exposed conditions.

**Table 3:** Effect of cinnamon on growth performance of broilers

Cinnamon form	Strains	Dosage (%)	Main findings	References
CBP	Lohmann	0.2	FBW (+ 2.4%) and FCE (+ 3.0%)	Sumi (2022)
CNE	Vencobb	0.1	BWG (+ 16.96%) and FCE (+ 16.1%)	Kour et al. (2025)
CEO	Cobb	1.5	FBW (+ 14.4%), BWG (+ 25.8%), FCE (+ 16.9%), and mortality (– 40%)	Wang et al. (2025)
CNP	Ross	0.045	BWG (+ 17%) and FCE (+ 18.7%)	Fathi et al. (2025c)

### Effects of cinnamon supplementation on carcass and meat quality

Cinnamon supplementation has been shown to influence carcass characteristics and meat quality parameters in chickens (Al-Abdullatif et al. 2023; Jimoh et al. 2024a; Khan et al. 2025). Park (2008) found that 3% CBP supplementation increased FBW, while 4% CBP improved meat water holding capacity (WHC). However, neither level affected the dressed percentage nor the weights of major cut-out parts (breast and thigh), which agree with the findings of Gaikwad and Fulpagare (2024) and Park (2008), both of which reported no effect on breast and thigh weights. In contrast, Khan et al. (2025) found that supplementation with 0.5 and 1.0% CBP improved dressing percentage, with the 1.0% level additionally increasing breast and thigh weights, while reducing abdominal fat content. On the same hand, Qaid et al. (2024) reported reduced abdominal fat content in broilers that received 0.2, 0.4, and 0.6% CBP supplementation. These variations in carcass traits support the observation by Sebola et al. (2015) that dosage level and chicken genotype influence carcass characteristics in chickens.

The observed improvements in dressing percentages and major cut-out weights may be ascribed to cinnamon’s bioactive compounds, primarily cinnamaldehyde, cinnamic acid, and eugenol, which have been found to stimulate digestive enzyme secretion and improve intestinal mucosa health (Ali et al. 2021). This could lead to better absorption of amino acids, the essential building blocks for muscle protein (Qaid et al. 2025). Furthermore, cinnamaldehyde acts as a natural activator of AMP-activated protein kinase (AMPK), a master regulator of cellular energy metabolism (Hwang et al. 2009). By stimulating AMPK phosphorylation, cinnamon can promote fatty acid oxidation in muscle cells and increases insulin-independent glucose uptake (Nishikai-Shen et al. 2022). This metabolic shift may favor fat utilization over abdominal fat deposition (Khan et al. 2025).

In broilers infested with *Eimeria tenella* oocysts, 0.2% cinnamon supplementation minimized the adverse impact of coccidiosis on carcass yield and cut-out weights. This protective effect is may be attributed to cinnamon’s antioxidant and anti-inflammatory properties, which reduces lipid peroxidation and pro-inflammatory cytokine release during acute phase of infection, thereby assisting to preserve muscle protein integrity and minimize catabolism of structural proteins. Jimoh et al. (2024b) demonstrated that supplementing drinking water with 300 ml/L CEO reduced drip loss and cooking loss in broiler breast muscle compared to the control broilers. This reduction in drip loss may be linked to the potential of polyphenols in cinnamon to stabilize the sarcolemma and cytoskeletal protein by inhibiting calpain-1 and caspase-3-activation, which may consequently delay extracellular matrix disruption and promote intracellular water retention (Ding et al. 2024). It can also act as an antioxidant that protects sulfhydryl () groups from oxidative degradation during heating. By preserving these groups and promoting beneficial protein cross-linking, it helps form a stronger protein matrix that traps moisture and minimizes drip and cooking loss. Qaid et al. (2024) confirmed these findings and further demonstrated that CBP supplementation reduces cooking shrinkage and shear force, resulting in improved meat tenderness. Improved meat tenderness is may be driven by cinnamon’s ability to accelerate post-mortem glycolysis, leading to a faster pH decline near the ultimate pH (5.8-6.0), which in turn increases myofibrillar fragmentation index (MFI) and reduces actomyosin cross-bridge formation. Additionally, the phenolic compounds chelate pro-oxidant metals, decreasing oxidative crossing linking of myosin heavy chains, thereby lowering shear force values and enhancing myofibrillar structural weakening during aging. Meat pH is an important indicator of quality, as it influences color, WHC, and shelf-life. Al-Abdullatif et al. (2023) reported that meat pH decreased with increasing dietary CBP levels, showing that cinnamon may accelerate post-mortem glycolysis and pH decline. When properly managed, this effect can be beneficial for meat preservation because after slaughter, muscle glycogen converts to lactic acid, driving a natural pH drop (Kiyimba et al. 2024). When this decline is well managed, it transitions the meat into an optimal state for longevity, appearance, and quality.

Meat color is a key factor influencing consumer acceptability and purchase decisions (Malheiros et al. 2025). Cinnamon supplementation has demonstrated variable effects on meat color parameters, depending on supplementation level and the form of cinnamon used (Park 2008; Qaid et al. 2024). A previous investigation by Park (2008) showed that breast meat from broilers fed 3% CBP was lighter in color compared to the control group fed diet without CBP supplementation. In contrast, Qaid et al. (2024) reported that supplementation with 0.6% CBP reduced meat yellowness at 24 h post-mortem, while having no effect on meat lightness (L*) or redness (a*). However, in the same study, dietary CBP supplementation levels of CBP at 0.2%, 0.4%, and 0.6% did not affect meat color parameters [L*, a*, and yellowness (b*)] at 15 min post-mortem. Similarly, Bosetti et al. (2020) noticed that supplementation of broiler diets with 0.01%, 0.02%, and 0.04% of essential oil (EO) containing a micro-encapsulated blend of 60% cinnamaldehyde, 30% carvacrol, and 10% carrier enhanced meat CP content and b* value compared to the group fed a diet without EO supplementation. This suggests the action of EO in improving broiler performance, corroborating the earlier results of Reis et al. (2018).

Lipid peroxidation is one of the factors that causes meat quality deterioration during storage, resulting in off-flavor, discoloration, and nutrient losses (Jimoh et al. 2024b). Broilers fed 0.3% CBP had reduced meat thiobarbituric acid reactive substances (TBARS) value compared to the control, i.e., without CBP supplementation (Park 2008; Sang-Oh et al. 2013). In a similar feeding study, Jimoh et al. (2024b) found that while the rate of lipid oxidation increased with storage time, CEO administration reduced lipid peroxidation compared to the control and increased GPx in breast meat on days 21 and 28. These findings indicate that cinnamon can extend the shelf life of broiler meat and can partly be explained by the antioxidant effect of cinnamon bioactive compounds (Elashry et al. 2026; Fathi and Zarrinkavyani 2026). Likewise, cinnamon supplementation has been demonstrated to alter fatty acid profile of broiler meat (Al-Abdullatif et al. 2023). Supplementation with 0.2% CBP reduced saturated fatty acids (FAs) and increased omega-3 FAs content in broiler breast meat compared to those fed 0.4 and 0.6% CBP (Qaid et al. 2022), suggesting that a low level of CBP can improve the lipid quality of broiler meat.

Consumer acceptance is the key measure of meat quality and is primarily evaluated by flavor, taste, juiciness, and overall palatability (O’Quinn et al. 2018). Gaikwad and Fulpagare (2024) found that dietary supplementation with 0.2% CBP enhanced sensory attributes of broiler meat, including flavor, taste, juiciness, and overall palatability when compared to the control. These findings are consistent with findings of Qaid et al. (2025), who also reported enhanced sensory attributes at the same supplementation level (0.2% CBP). The positive effects of cinnamon on meat sensory quality can be ascribed to antioxidant and antimicrobial activities of its bioactive compounds (Yang et al. 2021a; Elashry et al. 2026). These bioactive compounds, particularly cinnamaldehyde, exert their effects by scavenging free radicals, neutralizing hydroxyl radicals, and disrupting microbial cell membranes, thereby reducing lipid peroxidation and microbial growth in muscle tissue (Algothmi and Alshaya 2026). Reduced lipid peroxidation helps preserve meat juiciness by improving WHC (Park 2008). Furthermore, cinnamon may modulate post-mortem muscle metabolism, potentially leading to a more favorable pH and improved protein functionality, which collectively enhances tenderness, flavor stability, and overall eating quality (Ali et al. 2021).

### Effects on cinnamon supplementation on egg production and quality

Egg production plays an important role in global food security, human nutrition, and economic stability. It offers an affordable source of high-quality protein and essential micronutrients (Ma et al. 2026). As the global human population continues to increase, the poultry industry is expanding rapidly to meet the increasing demand for affordable, high-quality animal protein, especially in LMICs (OECD/FAO 2023).

Several studies have demonstrated positive effects of cinnamon supplementation on productive performance and egg quality in laying hens (Ghanem et al. 2021; Kianfar 2021; Bezahegn et al. 2025). However, results across studies are inconsistent. Yalçin and Denli (2021) and Bezahegn et al. (2025) reported that cinnamon inclusion in layer diets had no significant effect on feed intake and FCE. In contrast, Ghanem et al. (2021) and Kianfar (2021) observed improvements in feed intake and FCE in Lohmann Brown laying hens. These discrepant outcomes can be attributed to several interacting factors: (i) variations in cinnamon source, processing methods, harvesting seasons, extraction techniques, and environmental conditions, all which cause wide variability in the concentration of bioactive compounds and their biological activity; (ii) dosage level and diet composition; (iii) genetic strain and age of hens; and (iv) housing conditions, environmental stressors, and health status; and (v) differences in experimental duration. Short term trial may fail to capture adaptive responses in the gut microbiota, whereas longer supplementation periods allow for microbiota shifts and enzymatic acclimatization that translate into measurable performance gains.

These improvements in FCE may be attributed to two main mechanisms: (i) the stimulation of digestive enzymes and enhancement of intestinal mucosa by cinnamon’s bioactive compounds, and (2) modulation of the gut microbiota composition, which could lead to better nutrient utilization (Bento et al. 2013; O’Bryan et al. 2015). The bioavailability of cinnamon compounds can be influenced by gastric pH, intestinal transit time, and competitive microbial metabolism of these compounds by resident microbial populations. This variability might explain why some trials report null or marginal responses, while others show significant improvements.

Abo Ghanima et al. (2020) found increased egg production in ISA Brown layers (28–76 weeks old) fed diets supplemented with 0.02% CEO compared to the control. This finding is consistent with Bezahegn et al. (2025), who showed that supplementation with 0.75% and 1.75% CBP increased egg production by 8.97% and 8.59%, respectively in Lohmann brown laying hens, as well as egg mass, without affecting egg weight, or external egg quality. Similar improvements in productive performance have been reported by other researchers (Torki et al. 2015; Pakiza et al. 2020). Ghanem et al. (2021) further observed enhancements in egg production, egg weight, and egg mass, Haugh unit (HU), yolk index, and eggshell thickness in 24-week-old Lohmann Brown laying hens fed diets supplemented with 0.005%, 0.01%, and 0.015% CEO, with 0.015% being most effective. These beneficial effects are likely due to improved FCE, antioxidant effects, and better gut health promoted by cinnamon’s bioactive compounds.

Kianfar (2021) found that dietary supplementation with 1.0, 2.0, and 3.0% CBP improved egg production, egg mass, and egg storage durability in 60-week-old White leghorn laying hens without influencing egg weight. The improved storage durability may be linked to the antioxidant ability of cinnamaldehyde, the main bioactive compound in cinnamon bark, which helps prevent lipid peroxidation by increasing GPx activity and reducing oxidative stress markers (Abo Ghanima et al. 2020; Cimrin et al. 2020). Additionally, dietary cinnamaldehyde (0.01%) has been shown to improve yolk lipid profile by increasing oleic acid level and decreasing myristic acid content (Cimrin et al. 2020). This favorable shift may improve the nutritional quality of eggs for human consumption. The mechanism is likely mediated through activation of peroxisome proliferator-activated receptors (PPARs) and the modulation of fatty acid desaturase enzymes. Furthermore, the external application of CEO as a spray has also been reported to lower egg weight loss, air cell depth, yolk and albumen pH, and lipid peroxidation during storage (Vidyarthi et al. 2025).

Conversely, some studies reported non-significant effects of cinnamon supplementation on egg production and quality traits. Yalçin and Denli (2021) found that 0.5% CEO did not affect egg production and egg weight. Similarly, Soliman and Kamel (2020) reported that 1.0% cinnamon supplementation reduced yolk cholesterol and LDL content, as well as egg weight, but had no significant effects on egg production and egg mass. These non-significant effects of cinnamon supplementation on egg weight disagree with the findings of Ghanem et al. (2021) and Bezahegn et al. (2025). Such discrepancies could be due to differences in dosage level, form or type of cinnamon used, duration of supplementation, layer age, strain/genetics, basal diet composition, environmental or management factors.

Cinnamon supplementation has been shown to positively influence egg internal quality and reduce cholesterol content. Habeeb et al. (2020) found decreased yolk cholesterol and LDL levels, along with increased high-density lipoprotein (HDL) content in ISA Brown layers fed 0.35% cinnamon. The cholesterol-lowering effect could be attributed to the inhibition of hepatic 3-hydroxy-3-methylglutaryl-CoA reductase (HMGCR), the rate-limiting enzyme in cholesterol biosynthesis (Hu et al. 2022). This inhibition could lead to reduced cholesterol deposition in the egg (Torki et al. 2015). Furthermore, Abo Ghanima et al. (2020) and Bezahegn et al. (2025) reported that inclusion of cinnamon to layer diets positively influenced yolk quality (yolk index and color), albumen height, and HU, an index of albumen thickness and freshness. Cinnamaldehyde and other bioactive compounds in cinnamon are absorbed from the gut and can be deposited in the egg. The enhanced egg yolk color in layers on cinnamon supplementation may result from the accumulation of carotenoids (Soliman and Kamel 2020).

### Effect of cinnamon supplementation on hatchability

The use of PFAs rich in antioxidants to maintain physiological balance and prevent oxidative stress caused by high ambient temperature in tropical poultry production has been documented (He et al. 2018; Oke et al. 2021). In poultry trials, egg antioxidants have been shown to improve hatchability and enhance chick quality and performance during the early post-hatch period, which is critical for chick survival (Yang et al. 2021b). However, there are limited studies on the impacts of cinnamon supplementation on hatchability and chick quality. Kop Bozbay and Göneci (2023) reported that in-ovo feeding of cinnamon at 9 mg/egg improved hatchability and chick quality compared to the control (un-injected eggs). Similarly, Akosile et al. (2023) found that in-ovo administration of cinnamon extract at 2 mg/egg enhanced hatchability. The authors further noted that eggs injected with 2 mg of cinnamon extract exhibited higher hatchling weight than those injected with 4 mg of cinnamon extract. This observation aligns with the findings of El-Kholy et al. (2021), who reported that eggs injected with 0.5 mL of cinnamon had higher hatchling weights than uninjected eggs. These findings indicate that bioactive constituents in cinnamon provide oxidative protection against excess free radicals, which can negatively affect hatching success and chick quality (Oke et al. 2021).

### Limitations and safety concerns on the use of cinnamon as a PFA

Tropical environments, characterized by high ambient temperatures, elevated humidity, and seasonal fluctuations, present unique challenges that can influence the efficacy, safety, and practical application of cinnamon as a feed additive in chicken nutrition.

#### High-dose effects

One of the major limitations of cinnamon supplementation in chickens is the non-linear, often biphasic relationship between dosage and performance outcomes. While moderate levels typically improve performance, excessive supplementation can lead to adverse effects (Cimrin 2019). For example, CBP supplementation levels in broiler studies have varied widely, ranging from 0.015% to 5% in the diet (Park 2008; Tahami et al. 2023). This wide variation highlights the lack of standardized dosage guidelines and increases the risk of exceeding the optimal levels, which may compromise rather than enhance performance.

#### Palatability

Cinnamon supplementation rarely causes a strong depressive effect on feed intake at low or moderate doses. Most studies report improved or similar feed intake in chickens fed low to moderate levels of cinnamon compared to controls (Bezahegn et al. 2025; Khan et al. 2025; Qaid et al. 2025). However, at high doses (usually above 2–3% CBP), a depressive effect on feed intake is commonly observed (Cimrin 2019). This reduction is could be attributed to the strong, pungent, and bitter taste of cinnamon, which may reduce feed palatability.

#### Variability under environmental stress

Tropical conditions with ambient temperatures often exceeding 35 °C, combined with relative humidity above 70%, pose significant challenges to the stability of cinnamon’s bioactive compounds, particularly in essential oil form. The volatile nature of these compounds makes them vulnerable to thermal degradation during feed processing, such as pelleting, which exposes feed to temperatures of 70–90 °C. Although encapsulation technologies have been developed to improve thermal stability (e.g., encapsulated CEO product like NE-OFF), many producers in the tropical regions lack access to such advanced formulations (Kang et al. 2024). The efficacy of cinnamon as a heat stress mitigator in tropical poultry production is inconsistent and depends on several factors, such as particle size, delivery method, and thermal stress severity (Wang et al. 2025; Fathi and Zarrinkavyani 2026). Perera et al. (2024) reported that 0.5% CBP improved feed intake and BWG in heat-stressed broilers compared to the unsupplemented controls. However, efficacy appears to be dependent on the severity and duration of heat exposure.

### Safety concerns

Inclusion of cinnamaldehyde at 0.01% in layer diets significantly increased serum aspartate aminotransferase (AST), a biomarker of hepatocellular damage, and negatively affected FCE, egg weight, egg production, and eggshell quality (Cimrin 2019). Although cinnamaldehyde exhibits beneficial effects, by reducing blood sugar and cholesterol levels, these findings indicate that its margin of safety is relatively narrow, especially under tropical conditions where chickens may already experience elevated metabolic stress. The negative effects on egg production and quality appear to be downstream consequence of liver dysfunction. The liver plays central role in lipid metabolism, which is critical for yolk formation, as well as overall nutrient metabolism supporting egg production. When hepatocytes are damaged, these metabolic pathways may become compromised, potentially contributing to the observed decline in performance.

Similarly, Qaid et al. (2022) observed that while 0.2% CBP improved gut health, 0.6% supplementation increased cecal *E. coli* counts. Such paradoxical effects indicate that supra-optimal levels may disrupt cinnamon’s selective antimicrobial activity, potentially altering gut microbiota balance and favoring the growth of gut pathogenic bacteria. This disruption can compromise tight junctions between intestinal epithelial cells, allowing lipopolysaccharides (LPS), a potent endotoxin from Gram-negative bacteria to translocate from the intestinal lumen into the portal circulation (Cebi and Yilmaz 2025). According to Ghosh et al. (2020), the presence of LPS in portal circulation triggers a strong inflammatory response that can exacerbate hepatic stress and injury. Thus, the microbiota disturbances observed by Qaid et al. (2022) may represent an indirect contributing factor to the hepatic stress reported by Cimrin (2019). In effect, the liver is being “attacked” not only directly by the additive, but also indirectly by bacterial products originating from an imbalanced gut.

Although CEO is generally considered safe for consumers when used within approval dosage of 0.0025% in complete feed for poultry (Bampidis et al. 2022), concerns regarding potential residues in poultry products remains. Low levels of styrene (0.016–0.024%) have been detected in cinnamon bark oil; however, at proposed usage levels, styrene concentrations in complete feed are estimated to be only 1.2 × 10^− 7^ − 1.2 × 10^− 6^%, which is considered safe (Kang et al. 2024). Nevertheless, long-term residue studies are still needed in tropical poultry production systems, where degradation pathways may differ from those in temperate climates.

### Supplementation dosage levels for broilers and layers

Cinnamon is commercially available in several forms (bark powder, essential oil, extract, and nanoparticles), each with a distinct bioactive profile and bioavailability (Fathi et al. 2025a). As with other PFAs, its efficacy is highly dose-dependent. Suboptimal doses may yield negligible benefits, while excessive doses can produce negative effects. The reviewed studies suggest that best supplementation doses for broiler growth performance under normal conditions are 0.2–0.5% for CBP and 0.02–0.04% for cinnamon extract. Under heat-stress conditions, 1.5% CEO or 0.06% cinnamon nanoparticles appear more effective. The nanoparticle form consistently shows superior performance at lower supplementation levels, likely due to enhanced bioavailability. For carcass and meat quality traits in broilers, 0.2% CBP is likely best supplementation level for sensory attributes and overall meat quality, while 1.0% CBP appears more effective for improving carcass yield and cut-part weights. Supplementation of drinking water with 300 mg/L CEO yielded the best results for reducing cooking loss and improving shelf-life. In laying hens, 0.75-1.0% CBP or 0.015% CEO tends to improve productive performance, while 1.0–2.0% appear effective for enhancing egg quality and shelf life. Supplementation dosage with the best performance varies not only across product forms, but also according to bird type (broilers versus layers), age, strain, and environmental conditions. Therefore, the effective dose of cinnamon is dynamic and depends on the specific production goal (growth, carcass quality, egg production, or egg quality).

Importantly, these recommended doses should be interpreted as provisional guidelines rather than fixed prescriptions, because they are derived from studies conducted under specific experimental conditions that may not be universally reproducible. The actual supplementation level for any operation depends on multiple interacting variables, including cinnamon source, its form of preparation (powder, oil, extract, or nano-encapsulated), the production system; and prevailing environmental conditions. Further validation through well-designed, region-specific studies is strongly recommended. Such studies should account for local feed ingredients, water quality, climate patterns, and prevalent disease challenges. This will generate actionable, context-appropriate dose-response data to support precision supplementation strategies in the poultry industry.

### Cost-benefit, return on feed investment, and commercial feasibility

Feed accounts for about 65–70% of total production cost in poultry industry, making feed efficiency the single most critical driver of profitability in both broiler and layer operations (Al-Abdullatif et al. 2023), However, the commercial adoption of any feed additive depends on its ability to generate a positive return on investment through improved performance metrics that offset the cost of the additive. Therefore, any additive that improves FCE directly enhances the return on feed investment. Al-Abdullatif et al. (2023) evaluated the effects of four inclusion levels (0, 0.2, 0.4, and 0.6% CBP) in broiler diets. They observed that 0.2% treatment yielded the highest net revenue and benefit-cost ratio per kg of meat. Although broilers on cinnamon-supplemented diets had numerically higher feed costs than the control group, the superior BWG and FCE achieved with 0.2% CBP more than compensated for the additional expenditure.

Cinnamon’s commercial feasibility as a feed additive is supported by its relatively low cost as an herbal feed additive (Al-Abdullatif et al. 2023). When used at optimal dosages, it represents a cost-effective tool, particularly given the highly dose-dependent nature of its effects. This potential is further strengthened by additional benefits beyond simple growth promotion, including improved meat quality, reduced mortality rates (Kumar et al. 2018), and the opportunity to utilize low-cost feed ingredients.

### Cost comparisons with antibiotics or other phytogenic additives

The use of cinnamon as a feed additive in poultry production is an emerging area of research with promising benefits. In a direct comparison, broilers fed a diet with phytosome-conjugated cinnamaldehyde achieved significantly higher live weight and best FCE compared to a group fed an antibiotic bacitracin methylene disalicylate (BMD). This improvement translated into the highest profit per bird (Kondhare et al. 2024). Similarly, another study found that cinnamon powder improved economic efficiency compared to the antibiotic group (Kumar et al. 2018).

Results are more variable when cinnamon is compared with other phytogenic additives. El-Sharnobey et al. (2023) found that citric acid and spirulina were more effective than cinnamon oil (1 ml/kg feed) in improving economic efficiency. Citric acid had the added advantage of being cheaper and more readily available. In contrast, Hossain et al. (2014) reported that cinnamon, antibiotics BMD, and other herbs (black cumin and chilli) significantly improved FCE, BWG, and profitability, indicating that cinnamon can serve as a good alternative to antibiotics in broiler diets. Furthermore, Gaikwad and Fulpagare (2024) demonstrated that a combination of 2.0% cinnamon and 1.0% ginger effectively acted as a growth promoter and increased profit per bird. These findings suggest that cinnamon can be effectively combined with other phytogenics to optimize both performance and economic returns.

### Research gaps and future directions

Despite the growing body of evidence supporting cinnamon’s beneficial effects on poultry production, several important research gaps remain. The bioactive constituents of cinnamon differ considerably depending on species, plant part, soil conditions, and processing methods. Therefore, standardized products with quantified cinnamaldehyde content are essential for reproducible research and commercial applications. While cinnamon is generally regarded as safe, long-term studies on potential tissue residues of cinnamaldehyde and its metabolites are needed to ensure consumer safety. Additionally, more advanced studies employing transcriptomic, proteomic, and metabolomic methods are required to explain the molecular pathways through which cinnamon exerts its beneficial effects in broilers and laying hens.

Other critical gaps include the limited economic analyses on cinnamon supplementation, unexplained interaction between cinnamon and other dietary components, and the lack of dose-response trials for cinnamon nanoparticles and extracts in laying hens. There are also scanty studies on the impact of cinnamon on hatchability and more research is needed in this direction. Furthermore, inconsistent results across studies have been attributed to differences in dosage level, form or type of cinnamon used, duration of supplementation, bird age, strain, basal diet, and environmental conditions. These variations highlight the need for well-designed multi-factorial trials and meta-analyses to quantify the heterogeneity in responses of broilers and laying hens to cinnamon supplementation.

## Conclusion

This review shows that cinnamon is low in essential nutrients but high in bioactive compounds such as cinnamaldehyde, eugenol, and phenylpropanoids that can enhance performance in both broilers and laying hens through their antimicrobial, antioxidant, anti-inflammatory, cholesterol-lowering, and digestive enzyme-stimulating properties. This review suggests that in broilers, 0.2–0.5% CBP or 0.02–0.04% extract can improve growth performance, carcass yield, and meat quality while reducing abdominal fat content under thermoneutral conditions, while 0.03–0.06% CEO or 0.06% cinnamon nanoparticles showed superior performance under heat stress condition. In laying hens, supplementation with 0.75-1.0% CBP or 0.015% CEO showed improved FCE, reduced yolk cholesterol and lipid peroxidation, and enhanced egg quality. Overall, results indicate that cinnamon efficacy is dose dependent with excessive levels often proving detrimental.

For producer in low- and middle income-countries (LMICs) facing heat stress and rising feed costs, cinnamon offers an accessible phytogenic solution that aligns with growing consumer demand for antibiotic-free poultry products. However, practical adoption requires careful sourcing of consistent, high-quality cinnamon and proper dietary incorporation to maintain palatability and cost-effectiveness.

Despite these promising findings, the existing evidence has important limitations. Most studies reviewed were conducted under controlled experimental conditions with small sample sizes, short durations, and specific broiler or layer strains, which limit their generalizability to diverse commercial settings. The results vary widely due to differences in cinnamon species, processing methods, bioactive compound concentrations, basal diets, and environmental conditions. Long-term studies on safety, potential residues in meat and eggs, and interactions with other feed additives remain scarce. Moreover, large-scale commercial trials evaluating economic viability or performance consistency across various farm conditions are still lacking.

## Data Availability

Data will be made available on reasonable request.
